# Topological Constraints in Eukaryotic Genomes and How They Can Be Exploited to Improve Spatial Models of Chromosomes

**DOI:** 10.3389/fmolb.2019.00127

**Published:** 2019-11-15

**Authors:** Angelo Rosa, Marco Di Stefano, Cristian Micheletti

**Affiliations:** ^1^Scuola Internazionale Superiore di Studi Avanzati, Trieste, Italy; ^2^Centre Nacional d'Anàlisi Genòmica-Centre de Regulació Genòmica, Barcelona, Spain

**Keywords:** DNA and chromosomes, structural models, genomic entanglement, topological constraints, physical knots and links

## 1. Introduction

From viruses to eukaryotes, genomic DNA filaments are confined in spaces of linear dimension much smaller than their contour lengths. In bacteriophages, the μm-long genome is stored in 50 nm-wide viral capsids and the corresponding packing density is so high that viral DNA filaments that have little chance to be entangled in solution (knotting probability <3%) become almost certainly knotted (>95% probability) once confined inside the capsid (Rybenkov et al., 1993; Arsuaga et al., 2002; Marenduzzo et al., 2009, 2010). In humans, instead, the various cm-long chromosomes that make up the genome are kept inside 10 μm-wide nuclei (Alberts et al., 2014). Despite the major change of scale with respect to viruses, the volume fraction occupied by this eukaryotic genome is still large, about 10% (Rosa and Everaers, 2008).

These considerations pose several conundrums: How can chromosomal DNA be at the same time packed and yet accessible to the regulatory and transcriptional machineries? What is its typical degree of genomic entanglement and how much does it interfere with DNA transactions? To what extent are these aspects shaped by general passive physical mechanisms vs. active ones, e.g., involving topoisomerase enzymes?

## 2. Intra- and Inter-Chromosome Architecture

### 2.1. Phenomenology

Addressing these questions has proved challenging because of the wide range of length and time scales involved in genome architecture. Classical experimental tools provide details of chromosome architecture at two opposite scales (Marti-Renom and Mirny, 2011). At the smallest one (10 − 100 nm) X-ray crystallography revealed that DNA achieves local packing by wrapping around histones, while at the largest one (1 − 10 μm) fluorescence *in situ* hybridization (FISH) showed that each chromosome occupies a compact region of the nucleus, termed *territory* (Cremer and Cremer, 2001, 2010).

More recently, experimental breakthroughs such as super-resolution imaging, electron microscopy tomography plus selective labeling, and chromosome conformational capture (Hi-C) techniques have significantly extended our *multiscal*e knowledge of genome architecture (Dekker et al., 2002; Lieberman-Aiden et al., 2009; Boettiger et al., 2016; Ou et al., 2017; Bintu et al., 2018; Nir et al., 2018).

These and other advancements helped establish various results that foster the present discussion of genomic entanglement.

Regarding inter-chromosome organization we recall that:

- the positioning of chromosome territories correlates significantly with sequence-dependent properties of the underlying DNA [most notably, gene density (Bolzer et al., 2005)];
- the intermingling of different chromosomes is minimal and mostly restricted to the boundaries of the territories (Cremer and Cremer, 2001; Branco and Pombo, 2006).
  For intra-chromosome aspects we instead know that:
- on the scale of a few kilo-basepairs up to about 1 mega-basepair, chromosomes are organized into self-interacting regions, called *topologically-associating domains* or TADs (Dixon et al., 2012; Nora et al., 2012). On the tens of mega-basepairs scale, chromatin is organized into compartments of varying compactness depending on their functional and epigenomic state (Lieberman-Aiden et al., 2009; Wang et al., 2016);
- despite this variability, when averaged over chromosomes and experimental realizations, the mean contact probability of two chromosomal *loci* at genomic distance ℓ scales approximately as 〈*p*_*c*_(ℓ)〉 ~ ℓ^−1^ (Lieberman-Aiden et al., 2009), and the mean square separation scales as 〈*R*^2^(ℓ)〉 ~ ℓ^2/3^ (Sachs et al., 1995; Münkel et al., 1999).

### 2.2. Relating Genomic Architecture and Relaxation Dynamics With Polymer Physics

The interpretation of these experimental results has been aided by an intense theoretical and computational activity that demonstrated how salient genomic architecture properties can be reproduced by a broad range of polymer models, and hence are likely governed by general physical mechanisms (Mirny, 2011; Rosa and Zimmer, 2014; Bianco et al., 2017; Haddad et al., 2017; Jost et al., 2017; Tiana and Giorgetti, 2018). This applies in particular to the aforementioned properties (i–iv) which can be rationalized as manifestations of the topological constraints that rule the behavior of semi-dilute or dense polymer systems, particularly their relaxation time scales (Doi and Edwards, 1986).

In fact, a solution of initially disentangled chains of contour length *L*_*c*_ can reach the fully-mixed, homogeneous equilibrium state only via reptation, a slow and stochastic slithering-like motion with characteristic time scale equal to $\tau_{\text{rept}}≃\tau_{e}{\left(L_{c}/L_{e}\right)}^{3}$, where τ_*e*_ is a microscopic collision time and *L*_*e*_ is the typical contour length between entanglement points (De Gennes, 1971; Doi and Edwards, 1986).

Thus, based on this fundamental polymer physics result, it was estimated that the characteristic relaxation, or equilibration, time of mammalian chromosomes exceeds 100 years (Rosa and Everaers, 2008). The orders-of-magnitude difference between this time scale and the typical duration of the cell cycle (≈ 1 day) has several implications for genome organization, as it was realized even before Hi-C probing methods became available (Rosa and Everaers, 2008). It is clear, in fact, that mammalian chromosomes are never fully relaxed as they undergo the cyclic structural rearrangements from the separate compact rod-like mitotic architecture to the decondensed interphase one (Grosberg et al., 1993; Rosa and Everaers, 2008).

### 2.3. Implications for (Minimal) Intra- and Inter-chromosome Entanglement

From this standpoint, the emergence of chromosome territories is quantitatively explained as due to the kinetically trapped decondensation of the compact mitotic chromatin (Rosa and Everaers, 2008): interphase chromosomes retain the *memory* and *limited mutual overlap* of the earlier mitotic state, consistent with experimental results (Cremer and Cremer, 2001, 2010; Branco and Pombo, 2006). In addition, the ordered linear organization of the mitotic rods should also inform the intra-chromosomal architecture, making it more local than equilibrated polymers. This is consistent with the experimental fact that the effective scaling behavior of the contact probability with the genomic separation ℓ in interphase chromosomes has a more local character (~ℓ^−1^) than the one expected (~ℓ^−3/2^) for equilibrated polymers (Lieberman-Aiden et al., 2009). Intuitively, the same memory mechanism ought to facilitate the subsequent separation of interphase chromosomes and their recondensation upon re-entering the mitotic phase in the cell cycle (Rosa and Everaers, 2008).

For the present discussion, we stress that these out-of-equilibrium effects should impact not only the architecture but also the physical entanglement of eukaryotic genomes. In fact, mammalian chromosomes should be more unlinked (for the limited inter-chromosomal intermingling) and unknotted (for the enhanced intra-chromosomal local contacts) than at equilibrium. These heuristic conclusions are supported by various studies showing that the aforementioned scaling relationships obtained by FISH and Hi-C experiments can be ascribed to the topological constraints at play in solutions of unknotted and unlinked polymers (Khokhlov and Nechaev, 1985; Vettorel et al., 2009; Halverson et al., 2014;Rosa and Everaers, 2014).

### 2.4. Implications for Genomic Structural Modeling and Its Improvement

These considerations appear particularly relevant for the structural modeling of eukaryotic genomes based on phenomenological data, such as spatial proximity constraints, which are typically too sparse to pin down even coarse-grained models of interphase chromosomes (Lieberman-Aiden et al., 2009).

A key question is whether such structural models should *additionally* be informed by the notion that interphase chromosomes must originate and eventually return to the separate and condensed mitotic state.

**Figure 1:**
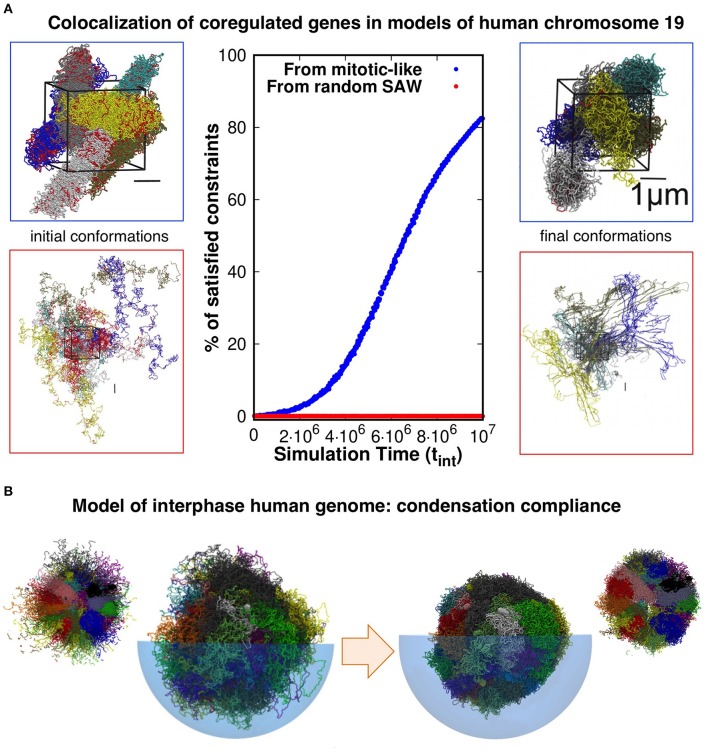
**(A)** Model conformations of human chromosome 19 (six copies, arranged in a periodic simulation box), described as self-avoiding chains of beads were reshaped by steered molecular dynamics (MD) simulations to promote the colocalization of pairs of *loci* that are significantly coregulated. Most (>80%) of the coregulated pairs were successfully brought into spatial proximity in simulations that started from relaxed solenoidal mitotic-like arrangements, while virtually no successful colocalization was observed for trajectories started from equilibrated, fully mixed, chromosomal arrangements. Adapted with permission from Di Stefano et al. (2013). **(B)** Model conformations of the entire-human genome, obtained by steered-MD colocalization of *loci* based on Hi-C data in Dixon et al. (2012) and Rao et al. (2014) could be successfully condensed with minimal hindrance from intra- or inter-chromosomal constraints, consistently with the expected reconfiguration compliance necessary for the interphase→mitotic transition. The smaller side pictures are cut-through views. Adapted with permission from Di Stefano et al. (2016).

Evidence presented in our earlier work help shed some light on the matter. With our co-workers, we considered a model system of six copies of human chromosome 19 in a cubic simulation box with periodic boundary conditions to explore the connection between coregulation and colocalization of genes (Di Stefano et al., 2013). Each copy was initially prepared as a mitotic-like conformation (Rosa and Everaers, 2008), consisting of a polymer filament forming a solenoidal pattern with rosette-like cross-section featuring chromatin loops of about 50 kilo-basepairs, see Figure 1A. We then used a molecular-dynamics steering protocol to bring in proximity pairs of intra-chromosomal *loci* that were known to be significantly co-regulated. Importantly, topological constraints were accounted for by avoiding unphysical chain crossings during the steering process.

Remarkably, and consistently with the gene kissing hypothesis (Cavalli, 2007), we found that most (>80%) pairs of significantly coregulated genes could indeed be colocalized in space within the contact range of 120nm and further showed that this colocalization compliance followed from the presence of gene cliques in the coregulatory network (Di Stefano et al., 2013).

Conversely, the same protocol applied to the same set of chains but initially prepared as generic self-avoiding random walks failed to give colocalization (Di Stefano et al., 2013). Physically, this happens because the intra- and inter-chain entanglements present in this system, which mimicks an artificial set of equilibrated chromosomes, were too numerous and conflicting to be successfully negotiated on a viable simulation time scale (see Figure 1A).

Further elements come from the genome-wide structural modeling of human chromosomes of Di Stefano et al. (2016). In this study too, the model chromosomes were initially prepared in mitotic-like states and were then steered to bring in proximity those pairs of *loci* that corresponded to significantly enhanced entries of two independent Hi-C datasets (Dixon et al., 2012; Rao et al., 2014). The architecture of the final conformations were, as expected, significantly changed by the steering protocol. Yet, as illustrated in Figure 1B, we verified that each model chromosome could be brought to a condensed compact shape as needed for the interphase-mitotic transition without significant hindrance from intra- or inter-chromosomal topological constraints (Di Stefano et al., 2016).

We note that the limitedly-entangled architecture of models of long eukaryotic chromosomes has emerged lately (Di Pierro et al., 2016) as the consequence of *microphase separation* of regions of different chromatin types (Jost et al., 2014) in a block co-polymer model with pair interactions tuned to reproduce the contact propensities of point (iv). The point is reinforced by studies on the yeast genome showing that knots and links have a generally low incidence especially in comparison to equivalent systems of equilibrated chains (Duan et al., 2010; Segal et al., 2014; Pouokam et al., 2019). Finally, besides the indication from structural models, other mechanisms such as loop extrusion have been advocated to be instrumental for maintaining a low degree of chromosomal entanglement (Racko et al., 2018; Orlandini et al., 2019).

To some inevitable extent though, physical entanglements are still expected to arise in eukaryotic chromosomes.

The recent work of Roca's lab showed that knots *do* occur in eukaryotic minichromosomes *in vivo*, for instance during transcription, due to transient accumulation of entanglement (Valdés et al., 2017, 2019). On broader scales, various knots (Siebert et al., 2017), and even links (Niewieczerzal et al., 2019), were found in model mouse chromosomes obtained from single cell Hi-C (Stevens et al., 2017). The genuineness of the entangled states was suggested by the systematic recurrence of certain knot types in independent instances of the reconstructed chromosomal structures (Siebert et al., 2017). These were obtained by imposing phenomenological constraints on an initially disconnected set of effective monomers, so we expect that a more defined knot spectrum could be obtained by using disentangled self-avoiding chains as the reference model.

## 3. Conclusions

To conclude, we have discussed experimental evidence and general physical mechanisms based on polymer theory that consistently point to an unusually low degree of entanglement expected in long eukaryotic chromosomes. Such property, which is arguably essential for the capability of chromosomes to reconfigure as needed at various stages of the cell cycle, appears important for genomic modeling too.

We argued that the structural modeling of long chromosomes can benefit, both for realism and computational efficiency, by starting off with disentangled self-avoiding chains, e.g., mitotic-like ones, because their plasticity makes it possible to accommodate a large number of phenomenological constraints in a physically-viable manner, i.e., without deformations involving intra- or inter-chain crossings.

The latter are, of course, possible in *in vivo* systems thanks to the action of topoisomerase enzymes. An important open question regards the extent to which these active mechanisms are involved in the shaping the overall intra- and inter-chromosome architecture. This point, we believe, can be significantly advanced in future studies with a tight synergy of experiments and models (Goloborodko et al., 2016; Jost et al., 2017; Valdés et al., 2019).

## Author Contributions

All authors listed have made a substantial, direct and intellectual contribution to the work, and approved it for publication.

### Conflict of Interest

The authors declare that the research was conducted in the absence of any commercial or financial relationships that could be construed as a potential conflict of interest.
